# Characterization and Expression Analysis of the bHLH Gene Family During Developmental Stages and Under Various Abiotic Stresses in *Sanghuangporus baumii*

**DOI:** 10.3390/genes16020184

**Published:** 2025-02-02

**Authors:** Ruipeng Liu, Tingting Sun, Pengyu Du, Zengcai Liu, Yawei Li, Xinyu Tong, Li Zou

**Affiliations:** 1 College of Forestry, Northeast Forestry University, Harbin 150040, China; liuruipeng@nefu.edu.cn (R.L.);; 2 College of Food Engineering, Harbin University, Harbin 150086, China

**Keywords:** *Sanghuangporus baumii*, *bHLH* gene, genome-wide analysis, abiotic stress, expression profile

## Abstract

Background: Basic helix–loop–helix (bHLH) transcription factors (TFs) widely exist in eukaryotic organisms and play a key role in plant growth and development in response to environmental stresses. *Sanghuangporus baumii*, an important medicinal mushroom known for its anticancer properties, has limited research on the bHLH gene family. Methods: This research utilized the genomic data from *S. baumii* to identify bHLH family members, and their gene structure, conserved motifs, and phylogenetic relationship were characterized. Additionally, we conducted an analysis of promoter cis-elements and predicted protein interaction networks. We also examined the expression profiles of bHLH genes during different developmental stages and in response to four abiotic stresses: heat, cold, oxidative stress, and heavy metal exposure. Finally, we overexpressed the candidate gene *SbbHLH3* in yeast to assess its tolerance to these different stress conditions. Results: A total of 12 *SbbHLH* genes were identified in *S. baumii*, and the members of the bHLH gene family displayed a variety of physicochemical characteristics, reflecting their diverse array of functions. Based on homology, the SbbHLH proteins are more closely related to those found in *Lentinula edodes* and *Pleurotus ostreatus*. The analysis of promoter cis-elements showed that *SbbHLH*s contain several elements associated with abiotic stress response, and a network prediction identified 28 bHLH-interacting proteins. Expression pattern analysis revealed that most *SbbHLH* genes exhibited a positive response to different developmental stages and abiotic stresses. Notably, the overexpression of *SbbHLH3* significantly enhanced stress tolerance in yeast. Conclusions: This study provides a comprehensive assessment of the bHLH family in *S. baumii*, delivering new genetic resources for breeding resistant varieties.

## 1. Introduction

*S. baumii*, a precious medicinal mushroom (previously classified as *Phellinus baumii* and *Inonotus baumii*), has been utilized as a traditional herbal remedy in China, Korea, Japan, and some other Asian countries for centuries [1]. This fungus typically grows on the trunk of *Syringa reticulata* and takes approximately 2 to 3 years to develop into yellow mature fruiting bodies [2]. In recent decades, *S. baumii* has garnered significant attention for its various bioactive compounds, including polysaccharides, triterpenoids, flavonoids, and phenolic compounds, which have important anticancer, antiviral, antioxidant, and anti-inflammatory activities [3,4,5,6]. Due to these properties, the wild resources of *S. baumii* are being depleted, so many researchers have explored artificial cultivation to increase the yield of this precious fungus [7]. However, *S. baumii* is susceptible to various abiotic stresses during its lifespan, such as oxidation and extreme temperatures, which lead to the weakening of its growth and development, as well as a significant decline in quality and yield.

Transcription factors (TFs), also known as trans-acting factors, play an important role in improving plant resistance to various environmental stresses [8,9]. TFs can specifically interact with cis-promoter elements of target genes to regulate specific physiological or biochemical processes in cells at the transcript level [10]. Structurally, a typical TF contains four functional domains: a DNA-binding domain, a transcription activation or repression domain, an oligomerization domain, and a nuclear localization domain. TF families, including members of the bHLH, MYB, bZIP, and other TF gene families, represent a significant portion of eukaryotic genomes, and their downstream target genes are extensively involved in numerous essential cellular signaling processes, such as differentiation, metabolism, development, and stress responses [11].

Basic helix–loop–helix (bHLH) proteins constitute one of the largest families of transcription factors and are widely distributed in plants, fungi, and animals [12]. The bHLH domain, which comprises approximately 50 to 60 conserved amino acids, features two primary components: the basic region and the helix–loop–helix (HLH) region. The basic amino acid region, located at the N-terminal and consisting of about 13 amino acids, is primarily responsible for DNA binding. Many bHLH domains recognize and bind to a specific DNA sequence known as the E-box (CANNTG). The HLH region, located at the C-terminal, consists of two α-helices separated by a loop. These α-helices form homo- or heterodimers with other α helices to stabilize the DNA interaction and facilitate transcription [13,14].

To date, most studies of bHLH genes have focused on plant species. The first bHLH gene identified in plants was in maize [15]. Since then, the bHLH gene family has been extensively researched in many plant species, including *Arabidopsis thaliana*, rice (*Oryza sativa*), sorghum (*Sorghum bicolor*), papaya (*Carica papaya*), Chinese white pear (*Pyrus bretschneideri*), carrot (*Daucus carota*), *Ginkgo biloba,* and *Rhododendron delavayi* [16,17,18,19,20,21,22]. In contrast, investigations into transcription factors in fungi, particularly mushrooms [23,24], are considerably fewer, with even less focus on bHLH factors. Despite the notable medicinal and economic importance of *S. baumii*, detailed reports on bHLH transcription factors in this mushroom are lacking, and the specific roles of bHLH genes in this species remain poorly understood. Therefore, it is of great significance to systematically study the bHLH family in *S. baumii*.

In this research, a total of 12 *bHLH* (*SbbHLH*) genes were identified from the reference genome of *S. baumii*, and phylogenetic analyses were conducted to explore the relationships among these genes. Additionally, we examined the physicochemical properties of the proteins, gene structure, motif composition, three-dimensional structure, and cis-elements in the promoter regions. We also analyzed the expression patterns of all 12 *bHLH* family members in *S. baumii* across various developmental stages and in response to abiotic stresses using quantitative real-time PCR (qRT-PCR). Notably, our findings demonstrated that the overexpression of *SbbHLH3* enhanced yeast resistance to abiotic stresses. This study may enhance the understanding of the functions of *SbbHLH* genes under abiotic stress conditions and provide a theoretical foundation for further functional research on the bHLH family in *S. baumii*.

## 2. Materials and Methods

### 2.1. Sample Preparation

*S. baumii* strain DL101 was collected from *S. reticulata* and the pure mycelial culture is preserved at the China Center for Type Culture Collection (CCTCC No. M2011137). The mycelium of *S. baumii* is maintained on potato dextrose agar (PDA) slants, stored at 4 °C, and subcultured every three months. Initially, a seed culture was prepared by incubating it in a rotary shaker incubator at 180 r/min and 25 °C for 8 to 10 days in liquid potato dextrose (PD) medium. Subsequently, a secondary experiment was conducted in 250 mL flasks containing 150 mL of PD medium, inoculated with 8% (*v*/*v*) of the seed culture. These flasks were shaken at 180 r/min at 25 °C for 5 days and then exposed to different stress conditions: 50 μM CdCl_2_ (Aladdin Biochemical Technology Co., Ltd., Shanghai, China), 10 mM H_2_O_2_ (Aladdin Biochemical Technology Co., Ltd., Shanghai, China), high temperature (40 °C), and low temperature (4 °C) for 24 to 48 h. Moreover, to examine the expression levels of *SbbHLH*s at different developmental stages of *S. baumii*, mycelium was collected at 3, 5, 7, and 9 days, as well as the primordia and young fruiting bodies. Each treatment was performed in triplicate. All samples were subsequently frozen and stored at −80 °C for later analysis.

### 2.2. Identification of bHLH Genes in the S. baumii Genome

The genome sequence data for *S. baumii* (NCBI accession number: ASM148141v2) were obtained from the NCBI database (https://www.ncbi.nlm.nih.gov/, accessed on 19 June 2023), and the hidden Markov model (HMM) file of bHLH domain (PF00010) was downloaded from the Pfam (http://pfam.xfam.org/, accessed on 20 June 2023) database. To identify SbbHLH gene family members, HMMER 3.0 software was adopted to search against the *S. baumii* protein sequences using default settings (E-value cut-off < 1 ×10^−5^); next, candidate bHLH sequences were further validated using SMART (http://smart.embl-heidelberg.de/, accessed on 21 June 2023) [25,26] and NCBI conserved domain searches. Moreover, we analyzed the physicochemical properties of the SbbHLH proteins, including coding sequence length, isoelectric point (pI), and molecular weight (MW), using the ExPASy Server (http://web.expasy.org/protparam/, accessed on 22 June 2023). The subcellular localization (SL) of the proteins was predicted with the online tool WoLF PSORT (https://www.genscript.com/wolf-psort.html?src=leftbar, accessed on 22 June 2023).

### 2.3. Characterization of the Structure and Motif of bHLHs

The exon–intron structures of *SbbHLH* genes were analyzed using the structure display server (GSDS 2.0, https://gsds.gao-lab.org/, accessed on 22 June 2023) [27]. Multiple sequence alignments of SbbHLH proteins were performed using ClustalW with default settings, followed by the construction of a phylogenetic tree based on 1000 bootstrap replicates via the neighbor-joining method using MEGA X 10.0.5 software [28]. The motifs of each deduced SbbHLH protein were identified by MEME (https://meme-suite.org/tools/meme, accessed on 23 June 2023) [29]. TBtools v2.136 software was used to visualize the NJ tree, gene structures, and the motifs of SbbHLH [30].

### 2.4. Phylogenetic Analysis and Classification of bHLH Gene Family

*Schizophyllum commune* is regarded as a model species among basidiomycetes, possessing 11 bHLH proteins. According to the classification of bHLHs in *S. commune* (ScbHLH), all identified *SbbHLH* genes were categorized into different groups. Multiple sequence alignments of ScbHLH and SbbHLH proteins were conducted using ClustalW, followed by the construction of a phylogenetic tree using the neighbor-joining (NJ) method with 1000 bootstrap replicates in MEGA X software. Moreover, the full-length amino acid sequences of bHLH proteins from *S.commune*, *Agaricus bisporus*, *Ophiocordyceps sinensis*, *P. ostreatus*, *Coprinopsis cinerea*, and *L. edodes*, obtained using a uniform method, were combined with the identified SbbHLH for phylogenetic analysis.

### 2.5. Homology Modeling of 3D SbbHLH Protein Structures

The secondary structure of the SbbHLH proteins was estimated utilizing SOPMA (https://npsa-prabi.ibcp.fr/cgi-bin/npsa_automat.pl?page=npsa_sopma.html, accessed on 23 June 2023). The tertiary structure of the SbbHLHs was constructed and visualized with the help of the SWISS-MODEL platform (https://www.expasy.org/resources/swiss-model, accessed on 24 June 2023).

### 2.6. Cis-Element Analyses in Promoter Regions of SbbHLH

To infer the potential function of *SbbHLH* genes, the upstream 2000 bp sequence from the transcription start site of each gene was extracted from the *S. baumii* genome, and the PlantCare website (https://bioinformatics.psb.ugent.be/webtools/plantcare/html/, accessed on 12 September 2024) was utilized for the prediction of cis-acting elements [31].

### 2.7. Protein–Protein Interaction Network Prediction

Protein–protein interaction networks were predicted using the STRING database (https://cn.string-db.org/, accessed on 19 September 2024) [32]. A total of 12 SbbHLH protein sequences served as queries, with orthologs from *Phellinus noxius* (as *S. baumii* was formerly classified under the genus *Phellinus*) selected as references. The interaction network for SbbHLH proteins was constructed utilizing the proteins with the highest scores from BLAST analysis.

### 2.8. Total RNA Extraction, cDNA Reverse Transcription, and qRT-PCR Analysis

All twelve *SbbHLH* genes were analyzed by qRT-PCR at different developmental stages and under different stress conditions. Total RNA was extracted using the RNAprep Pure plant kit (TIANGEN BIOTECH Co., Ltd., Beijing, China). For reverse transcription, 1 μg of RNA was used as the template to synthesize the first-strand cDNA with the PrimeScript™ RT reagent Kit containing gDNA Eraser (Takara Biomedical Technology Co., Ltd., Dalian, China). The primers for qRT-PCR were designed with Primer Premier 5.0 software (Table S1). The procedure for qRT-PCR was described in previous studies, with the α-tubulin gene used as an internal control [33]. Each experiment was performed in triplicate, and relative gene expression levels were calculated using the 2^−(ΔΔCt)^ method [34].

### 2.9. Cloning of the SbbHLH3 Gene

Based on the *SbbHLH3* nucleic acid sequence obtained, primers were designed with Primer Premier 5.0 software (Table S1) using *S. baumii* cDNA as a template and high-fidelity enzyme for *SbbHLH3* cloning. The purified PCR products were subsequently ligated into the pMD18-T cloning vector (Takara Biomedical Technology Co., Ltd., Dalian, China) for amplification. The sequencing results were verified to match the genomic sequencing data, and these confirmed sequences were then utilized to construct the expression vector.

### 2.10. Plasmid Construction and Overexpression Analysis of SbbHLH3 in Yeast

Firstly, the pYES2 vector was digested by the single enzyme (*Hin*dⅢ, Takara Biomedical Technology Co., Ltd., Dalian, China), and the *SbbHLH3* coding sequence was then ligated into the pYES2 vector using MS FastCloning MultiS kit plus (Zhaokui Biomedical Technology Co., Ltd., Beijing, China). The recombinant vector was then transferred into *Escherichia coli* and sent to Harbin Biotech for sequencing (Genesoul Biotech, Harbin, China). The correctly sequenced recombinant vector was designated as pYES2-SbbHLH3. Both the recombinant vector and the empty pYES2 vector were transformed into INVScI yeast (Weidi BIOTECH Co., Ltd., Shanghai, China) following the manufacturer’s protocol. The transformed yeast cells were then cultured on selective SD-Ura medium (Weidi BIOTECH Co., Ltd., Shanghai, China) at 30 °C for a period of 24 to 48 h. To confirm the accuracy of the sequence, PCR verification was conducted after selecting single colonies, using the primers listed in Table S1 (pYES2-T7/pYES2-CYC1).

To verify the tolerance of *SbbHLH3* gene to abiotic stresses, the transformed pYES2-SbbHLH3 yeast and the control yeast (harboring only the pYES2 plasmids) were first incubated in SD-Ura, and then transferred to SC-Ura induction medium containing 2% galactose and incubated at 30 °C for 24 h. Subsequently, the optical density (OD_600_) was adjusted to 1.0, and the supernatant was discarded after centrifugation. To detect the tolerance of oxidation and heavy metal resistance, the yeast cells were resuspended in 1 mL of SD-Ura medium containing H_2_O_2_ solution with a final concentration of 100 mmol L^−1^, 200 mmol L^−1^, and 400 mmol L^−1^, respectively, as well as cadmium chloride solution with a final concentration of 200 μmol L^−1^, 400 μmol L^−1^, and 800 μmol L^−1^. The cultures were then incubated at 30 °C for 24 h. In addition, for freezing and heat stress evaluation, the yeast cells were resuspended in SD-Ura liquid medium and incubated at −20 °C for 24 h and at 50 °C in a water bath for 2 h, respectively. After treatment, the cells were serially diluted (10^0^, 10^−1^, 10^−2^, 10^−3^, and 10^−4^), and 2 µL drops of the diluted samples were spotted onto the SD-Ura agar medium. The results were evaluated after 2 days of incubation at 30 °C. All experiments were conducted in triplicate.

## 3. Results

### 3.1. Identification and Classification of bHLH Genes in S. baumii

A total of 12 bHLH transcription factors were identified in the *S. baumii* genome (Table S2). To facilitate their identification, they were designated SbbHLH1 through SbbHLH12. The predicted protein lengths of the SbbHLH ranged from 244 (SbbHLH6) to 1254 (SbbHLH8) amino acids. The proteins’ theoretical isoelectric point (pI) values ranged from 5.50 (SbbHLH11) to 9.55 (SbbHLH2), while their estimated molecular weights (MW) were between 26.26 (SbbHLH6) and 137.86 kDa (SbbHLH8). According to subcellular location prediction, most proteins worked in the nucleus, except for SbbHLH5 and SbbHLH8, which were predicted to be located in the extracellular space and mitochondrial compartment, respectively (Table 1). The bHLH proteins from *S. baumii* were classified as acidic due to their average pI being 6.76. We discovered that all family members are hydrophilic proteins based on Grand Average of Hydropathicity (GRAVY) assessments. Members of the bHLH gene family exhibit a range of physicochemical properties, reflecting their diverse array of functions.

### 3.2. Gene Structure and Conserved Motif Analysis of SbbHLH

**Figure 1:**
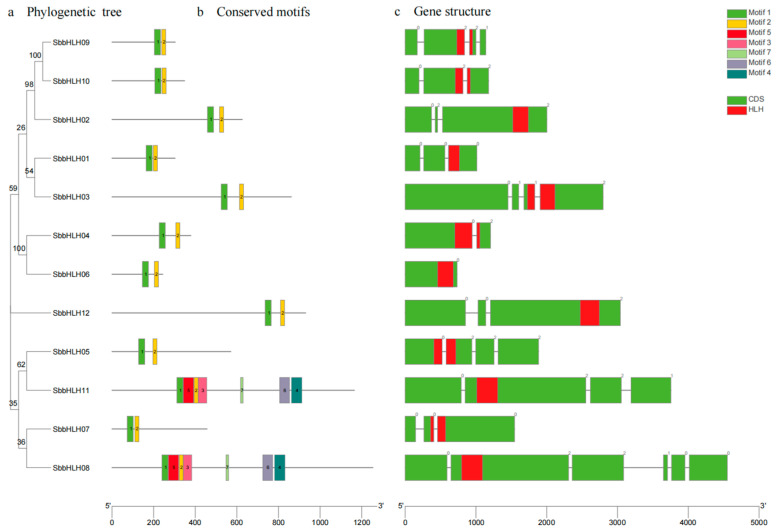
Characterization of *S. baumii* bHLH genes. (**a**) Phylogenetic analysis of SbbHLH proteins from *S. baumii*. (**b**) Conserved motifs of SbbHLH proteins. (**c**) Gene structures of SbbHLH genes. Introns are shown as gray lines.

To gain a deeper understanding of the structural components of the *SbbHLH* genes, we conducted an analysis of their exon–intron structures. The number of introns varied among the *SbbHLH* genes, ranging from 0 to 5 (Figure 1a,c). The majority of *SbbHLH* genes (41.67%) contained two introns, while the other genes predominantly had three introns. Notably, SbbHLH8 contained the highest number of introns, totaling five, whereas SbbHLH6 was found to have no introns.

Additionally, we examined the sequence characteristics of the bHLH family members in *S. baumii* by analyzing the conserved motifs of the 12 SbbHLH proteins using the MEME program. We identified a total of seven specific conserved motifs (Motif 1–7) (Table S3). As shown in Figure 1b, nearly all SbbHLH proteins exhibited the conserved Motif 1 and Motif 2, which were located closely together within the bHLH proteins. Interestingly, we observed that certain motifs were exclusive to specific SbbHLH proteins. For instance, Motifs 3–7 were uniquely present in SbbHLH8 and SbbHLH11, suggesting that these two genes may have distinct functions compared to the other SbbHLH members.

### 3.3. Multiple Sequence Alignment, Phylogenetic Analysis, and Classification of SbbHLH Genes

**Figure 2:**
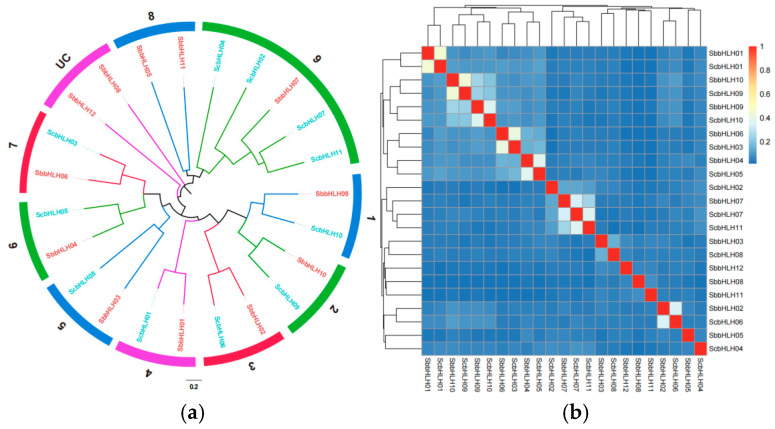
The phylogenetic tree among bHLH domains of *S. baumii* and *S. commune*. (**a**) The phylogenetic tree was derived using the NJ method in MEGA X. (**b**) Heatmap of sequence similarity among members of the bHLHs of *S. baumii* and *S. commune*.

For a better understanding of the evolutionary relationship between *S. baumii* and model basidiomycete Schizophyllum commune, multiple sequence alignment was conducted using ClustalW, focusing on the amino acid sequences of 12 SbbHLH and 11 ScbHLH proteins (Tables S2 and S4). Subsequently, a phylogenetic tree was constructed utilizing the neighbor-joining (NJ) method with a bootstrap value of 1000, implemented in Mega X (Figure 2a). The phylogenetic analysis revealed that the 23 bHLH genes were categorized into nine distinct clades, along with one unclassified group (UC) that encompassed two SbbHLH proteins (SbbHLH8 and SbbHLH12). Notably, none of the ScbHLH proteins were placed in subfamily 8, which exclusively contained two SbbHLH proteins (SbbHLH5 and SbbHLH11), suggesting a potential novel evolutionary pathway for *S. baumii*. Among the identified subfamilies, subfamily 9 was the most populous, comprising five bHLH members, while the remaining subfamilies each contained two members.

The sequence similarity of the bHLH gene family members from the two species was assessed and visualized using a heatmap (Figure 2b). Several SbbHLHs were tightly clustered with the ScbHLHs (bootstrap support ≥ 70). These SbbHLHs are likely orthologous to the ScbHLHs and may have analogous functions.

### 3.4. Evolutionary Analysis of bHLH in S. baumii and Several Different Macrofungus Species

**Figure 3:**
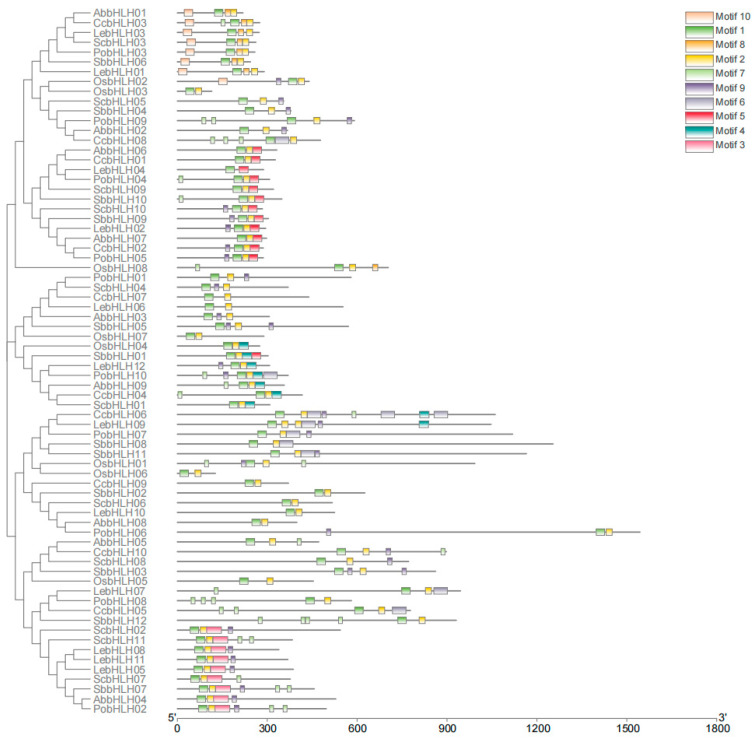
Phylogenetic relationship and motif composition of the bHLH proteins from *S. baumii* with six different macrofungus species (*S. commune*, *A. bisporus*, *O. sinensis*, *P. ostreatus*, *C. cinerea*, and *L. edodes*).

To investigate the evolutionary relationships of bHLH proteins in *S. baumii* and six other macrofungus species (*S. commune*, *A. bisporus*, *O. sinensis*, *P. ostreatus*, *C. cinerea*, and *L. edodes*), we constructed an unrooted neighbor-joining tree incorporating 10 conserved motifs identified by the MEME web server (Figure 3, Table S4). The distribution of SbbHLHs in the phylogenetic tree is notably widespread. With the exception of LebHLH4, all other proteins from the six macrofungi analyzed contained Motifs 1 and 2. Moreover, we observed that the bHLH proteins from *L. edodes*, *P. ostreatus*, and *S. baumii* clustered on the same phylogenetic branch, indicating a generally similar motif composition among them. This suggests a closer evolutionary relationship between SbbHLH proteins and those found in *L. edodes* and *P. ostreatus*. Overall, individuals within the same subfamily displayed comparable patterns of conserved motifs, and supporting the reliability of the phylogenetic analysis.

### 3.5. Cis-Regulatory Element Analysis of SbbHLH Gene Promoters

**Figure 4:**
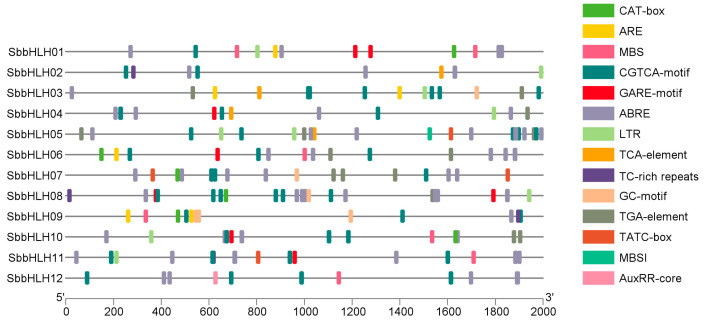
Cis-acting element analysis of SbbHLH promoters.

To better understand the regulatory mechanisms of the SbbHLH gene family in response to abiotic stress, the cis-elements in the promoter region were analyzed using the online database PlantCARE, and the redundant and irrelevant sequences were manually removed before TBtools was used to visualize the findings. As illustrated in Figure 4, the SbbHLH promoter harbors various stress-responsive elements, including TC-rich repeats (involved in defense and stress responsiveness), ARE (anaerobic response element), LTR (low-temperature responsiveness element), and MBS (MYB binding site associated with drought inducibility). Furthermore, the promoter regions also contained several hormone-responsive and growth- and development-related elements, such as the MeJA/CGTCA-motif, abscisic acid/ABRE, salicylic acid/TCA-element, gibberellin/GARE-motif, and auxin/AuxRR-core (Figure 4). Therefore, members of the bHLH gene family in *S. baumii* are likely to be involved in multiple responses (e.g., stress response) by regulating the upstream elements of transcription factors.

### 3.6. Analysis of the 3D Structure of SbbHLH Proteins

The three-dimensional (3D) architecture of proteins plays a crucial role in understanding their functions. Consequently, we performed a 3D structural analysis of all 12 SbbHLH proteins using SWISS-MODEL. The findings revealed a significant similarity among several protein pairs, specifically SbbHLH3/4, SbbHLH9/10, and SbbHLH8/11 (Figure S1). By integrating the 3D structures of the SbbHLH proteins, we can gain a deeper insight into the similarities and differences among the family members. This understanding will serve as a valuable reference for further investigations into the functions of SbbHLH proteins in *S. baumii*.

### 3.7. In Silico Protein–Protein Interaction of SbbHLH Proteins

**Figure 5:**
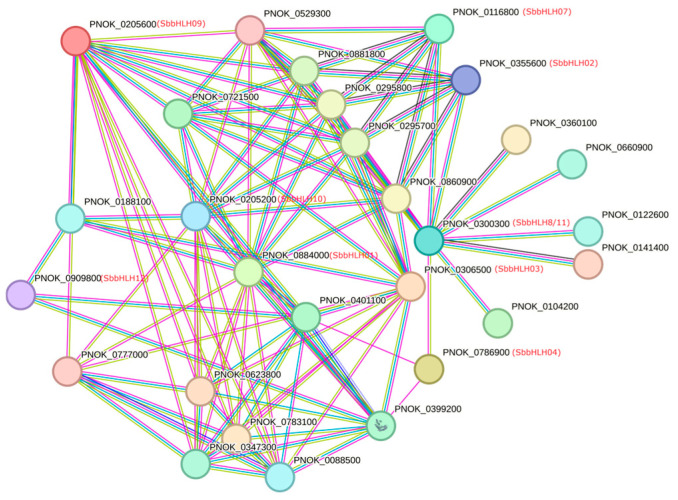
Prediction of SbbHLH-interacting protein networks in *S. baumii*.

To investigate the relationships between SbbHLHs and other proteins, a protein interaction network was constructed by mapping SbbHLHs to homologs from *P. noxius* (as *S. baumii* was previously classified under the genus Phellinus) using the STRING online database (Figure 5). The results revealed that 10 SbbHLHs interacted with each other, while the remaining SbbHLH5 and SbbHLH6 showed no interaction with any bHLH proteins. In the network, there were 28 bHLH-interacting proteins, including low temperature requirement protein, steroid regulatory element binding cleavage-activating protein, WD40 domain-containing protein, membrane-bound transcription factor site-2 protease-like protein, and PAS domain-containing protein. These findings provide a valuable reference for further functional studies of the bHLH gene family in *S. baumii*.

### 3.8. Expression of SbbHLH Genes at Different Developmental Stages

**Figure 6:**
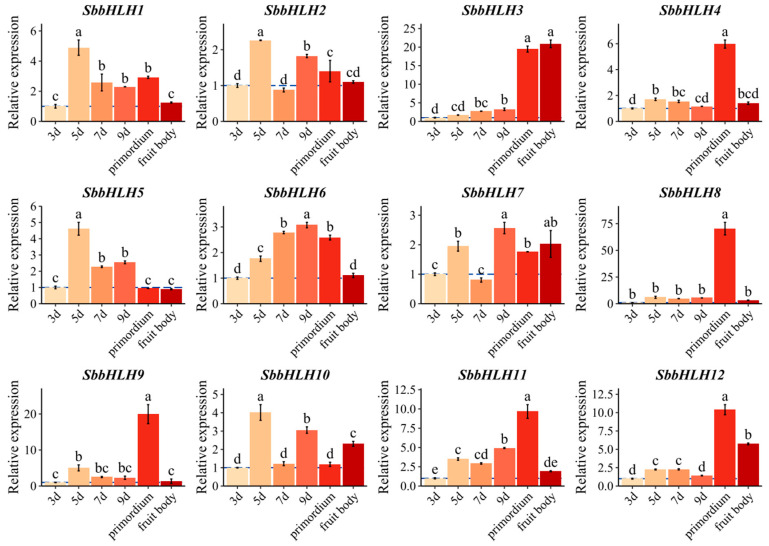
Gene expression of 12 *SbbHLH* genes determined by qRT-PCR during different developmental stages of *S. baumii*. (The data shown are mycelium cultured for 3~9 days: 3d, 5d, 7d, and 9d; primordium; and fruit body.) The error bars represent the standard error of the mean of three technical replicates. Lowercase letters indicate significant differences among treatment means (α = 0.05, LSD). The dashed line indicates that the relative expression level of the gene reaches “1”, making it easy to compare with the control.

To investigate the role of bHLH family genes at different developmental stages of *S. baumii*, we assessed the relative expression levels of all 12 *SbbHLH* genes in mycelium cultured for 3 to 9 days, as well as in primordia and fruiting bodies, using qRT-PCR. Our analysis revealed distinct expression patterns for the *SbbHLH* genes across the different developmental stages, indicating that these genes may fulfill diverse regulatory functions. Notably, four genes (*SbbHLH1*, *SbbHLH2*, *SbbHLH5*, and *SbbHLH10*) exhibited peak transcript levels in mycelium cultured for 5 days, while two genes (*SbbHLH6* and *SbbHLH7*) reached their highest expression in mycelium cultured for 9 days. This suggests a potential involvement of these genes in mycelial development in *S. baumii*. In contrast, *SbbHLH3*, *SbbHLH4*, *SbbHLH8*, *SbbHLH9*, *SbbHLH11*, and *SbbHLH12* displayed elevated expression during the primordia and fruiting body stages, leading us to hypothesize that these six SbbHLH genes may be more closely linked to the development of primordia and fruiting bodies (Figure 6).

### 3.9. Expression Patterns of SbbHLH Genes in Response to Different Stress Treatments

**Figure 7:**
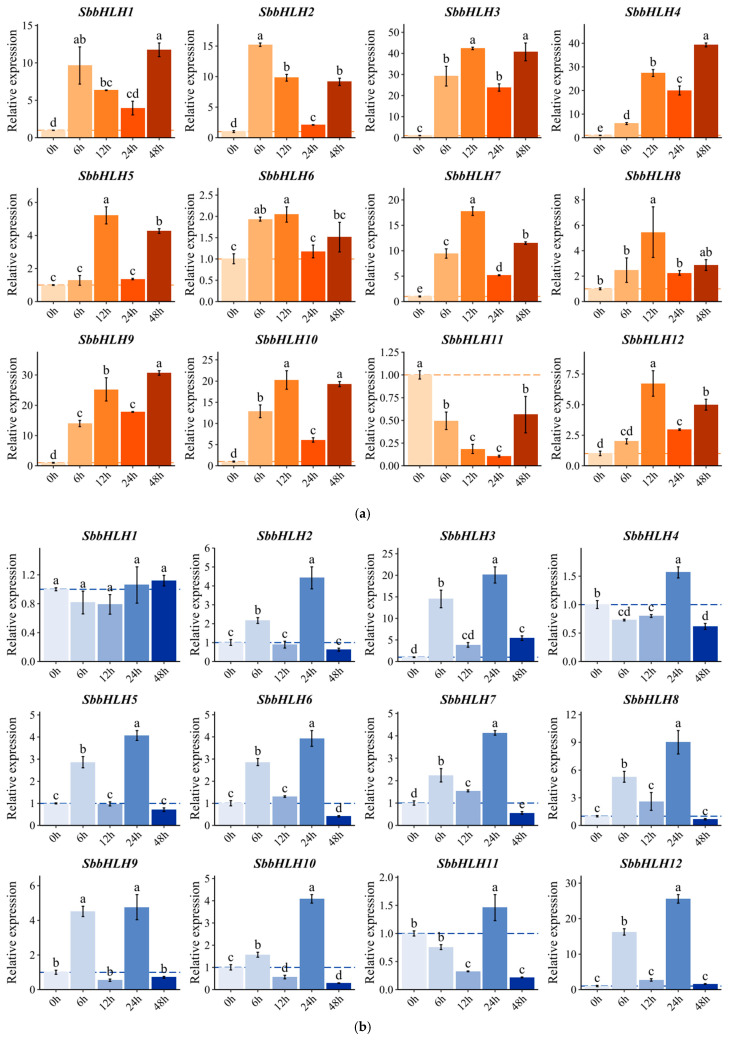

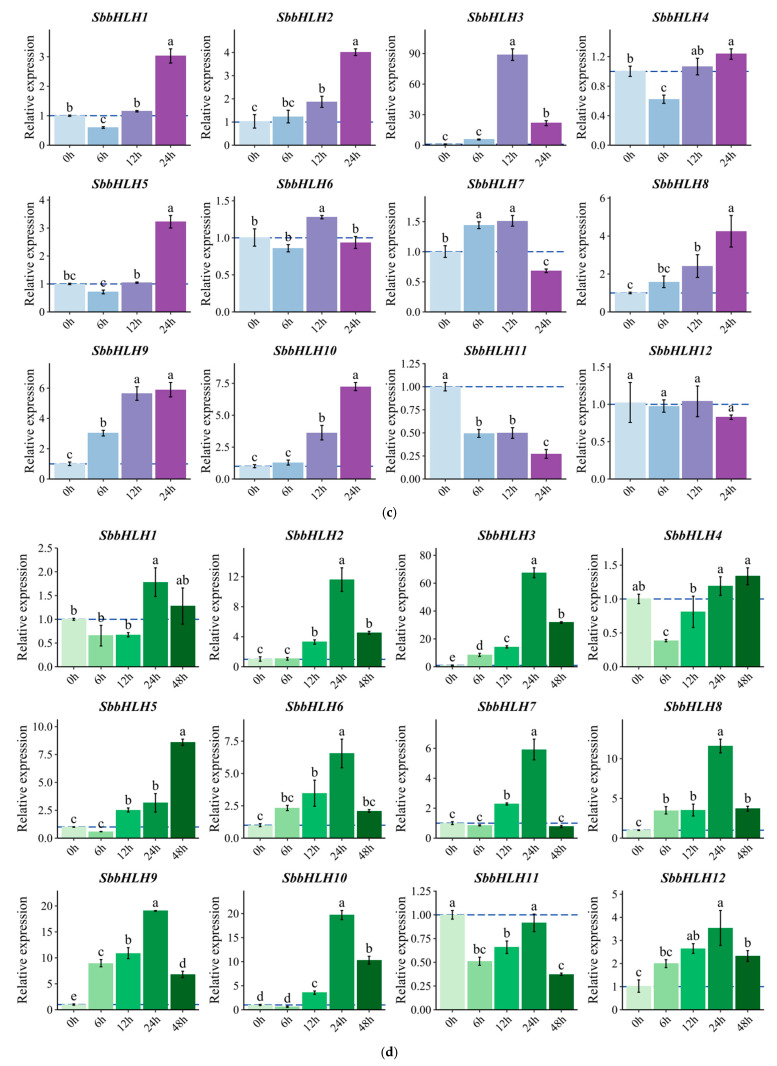
The expression profile of 12 *SbbHLH* genes in *S. baumii* under abiotic stresses as evaluated by qRT-PCR: (**a**) heat treatment; (**b**) cold treatment; (**c**) H_2_O_2_ treatment; and (**d**) CdCl_2_ treatment. The error bars represent the standard error of the mean of three technical replicates. Lowercase letters indicate significant differences among treatment means (α = 0.05, LSD). The dashed line indicates that the relative expression level of the gene reaches “1”, making it easy to compare with the control.

To evaluate the influence of different abiotic stresses on the expression of *SbbHLH* genes, we conducted a qRT-PCR analysis of 12 *SbbHLH* members subjected to four types of abiotic stress: H_2_O_2_, CdCl_2_, heat, and cold treatments. The results revealed diverse expression patterns among the *SbbHLH* genes. Most of the *SbbHLH* genes exhibited significant upregulation under heat stress, with the exception of *SbbHLH11* (Figure 7a). On the contrary, fewer *SbbHLH* genes were upregulated in response to cold stress compared to heat stress, mainly including *SbbHLH3*, *SbbHLH8*, and *SbbHLH12* (Figure 7b). Additionally, the expression levels of certain *SbbHLH* genes varied significantly over time depending on the specific stress treatment. For instance, under CdCl_2_ stress, the expression of *SbbHLH3* and *SbbHLH9* was initially elevated but then declined (Figure 7d). Interestingly, some genes displayed contrasting expression patterns under different stress conditions; for example, *SbbHLH12*, was upregulated in response to heat and CdCl_2_ treatments, yet downregulated under H_2_O_2_ exposure (Figure 7a,c,d). Notably, *SbbHLH3* showed a particularly robust response across the various abiotic stresses. Under heat, cold, H_2_O_2_, and CdCl_2_ treatments, *SbbHLH3* reached the highest transcript levels (41.7-fold, 20.3-fold, 88.9-fold, and 67.5-fold, respectively) at 12 or 24 h post-induction when compared to the control. Therefore, *SbbHLH3* appears to play a crucial role in mediating abiotic stress responses in *S. baumii* and needs further study.

### 3.10. Overexpression of SbbHLH3 Enhances Yeast Tolerance to Abiotic Stress

**Figure 8:**
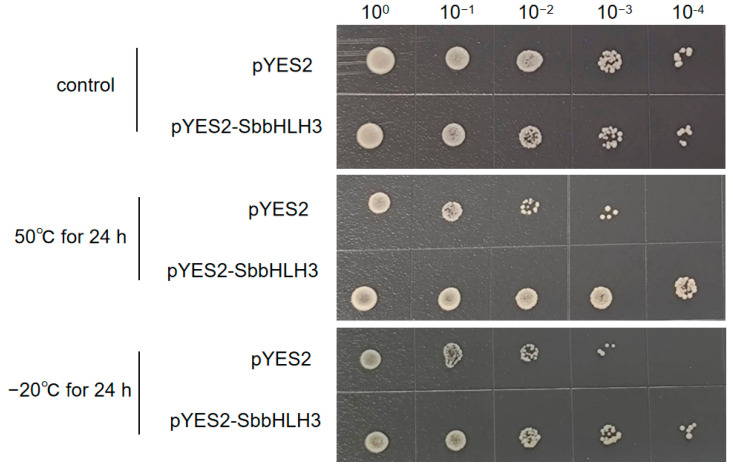
Effects of SbbHLH3 expression on the survival of *S. cerevisiae* strains with heat or cold or no treatment.

To explore the roles of bHLH family genes in the abiotic stress response of *S. baumii*, SbbHLH3 was selected for preliminary functional validation based on qRT-PCR expression levels. The coding sequence of SbbHLH3 was cloned into pYES2 plasmids and subsequently transformed into *Saccharomyces cerevisiae* strains INVSC1. The successful recombinant yeast strains (pYES2-Sb-bHLH3) were confirmed through PCR analysis (Figure S2). Both the positive and control INVSC1 yeast cells were cultured on SD-Ura with different abiotic stress treatments. As shown in Figure 8, under non-stress conditions, there was no notable difference in growth between the control (pYES2) and the transformed yeast (pYES2-Sb-bHLH3), indicating that the overexpression of SbbHLH3 does not affect yeast growth. In contrast, after exposure to heat (50 °C) or cold (−20 °C) stress, the transformed yeast (pYES2-Sb-bHLH3) exhibited superior growth compared to the empty vector control (pYES2).

**Figure 9:**
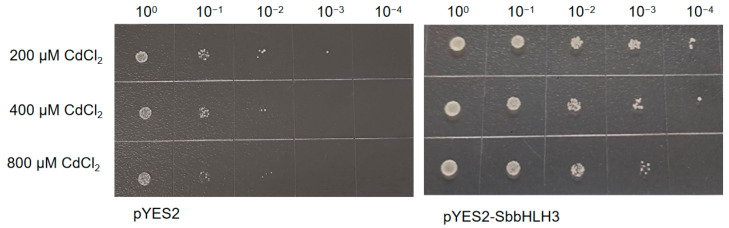
Effects of SbbHLH3 expression on the survival of *S. cerevisiae* strains supplemented with different concentrations of CdCl_2_.

**Figure 10:**
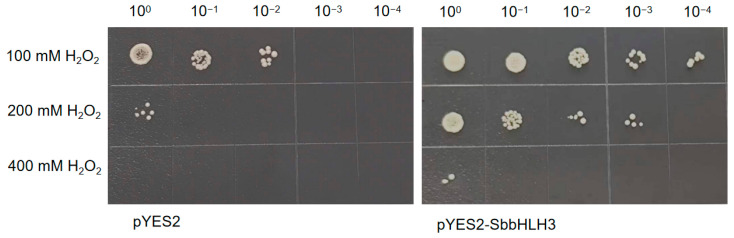
Effects of SbbHLH3 expression on the survival of *S. cerevisiae* strains supplemented with different concentrations of H_2_O_2_.

Furthermore, the pYES2-Sb-bHLH3 yeast strain and the control yeast were cultured under varying concentrations of CdCl_2_ and H_2_O_2_ stress. The results showed that both CdCl_2_ and H_2_O_2_ stress significantly affected yeast cell growth. However, it was evident that the growth of the pYES2-Sb-bHLH3 yeast strain was notably superior to that of the control (Figure 9 and Figure 10). For example, under 200 mM H_2_O_2_ stress, the pYES2 control yeast cells did not exhibit any growth after dilution, whereas the pYES2-Sb-bHLH3 yeast continued to grow even after a 1000-fold dilution. This suggests that the overexpression of SbbHLH3 enhances the yeast’s resistance to oxidative stress. In summary, these results indicate that *SbbHLH3* may play a beneficial role in the response of fungi to abiotic stress.

## 4. Discussion

The bHLH gene family has been identified as the second-largest transcription factor family in plants, followed by MYB, and plays a crucial role in various signaling pathways related to growth, development, and stress responses [35]. To date, numerous bHLH genes have been identified in different plant species. Specifically, 164 bHLH TFs were identified in *Arabidopsis*, 174 in *S. bicolor*, 180 in rice, 190 in tobacco, 191 in grapes, 146 in carrot, and 208 in maize [16,17,21,36,37]. In this study, we identified 12 bHLH genes in *S. baumii*. The size of the bHLH gene family in *S. baumii* (12) is smaller compared to those in plants but is comparable to other fungi, such as 12 in *L. edodes*, 11 in *S. commune*, 10 in *P. ostreatus*, 9 in *A. bisporus*, 10 in *C. cinerea*, and 8 in *O. sinensis*, as determined from their genomic data (Table S4). These findings suggest that the large bHLH gene family in plants has experienced considerable expansion, likely accompanied by subfunctionalization, neofunctionalization, and changes in gene expression patterns and protein–protein interactions.

The protein motif and gene structure analysis indicated that various members of the same subfamily likely share common evolutionary origins and may have similar physiological roles. The number of exons/introns in SbbHLH family members varied from 0 to 5, resembling findings from previous studies. This indicates that during the evolution of *S. baumii*, the bHLH gene exons and introns underwent processes of loss or gain (Figure 1c). Through phylogenetic analysis with *S. commune*, we categorized the SbbHLH family proteins into nine distinct subfamilies (Figure 2a). Subcellular localization studies revealed that the majority of SbbHLHs were localized in the cell nucleus (Table 1), aligning with the findings related to bHLH family genes in other organisms [17,38].

The bHLHs can form homodimers or heterodimers and bind to specific cis-acting elements on target genes [39]. In this study, we examined the cis-regulatory elements of *SbbHLH* gene promoters and identified conserved regulatory modules that are characteristic of various members involved in stress responsiveness, as well as growth- and development-related elements in the promoter regions. Notable motifs identified include TC-rich repeats, ARE, LTR, and MBS motifs. The findings suggest that SbbHLHs may play a role in growth and stress resistance in *S. baumii*. Additionally, predicted protein interaction networks provide a theoretical framework for exploring the physiological roles of different bHLH family proteins. The physical interactions among proteins within the network imply that SbbHLHs could participate in a range of cellular processes. However, SbbHLH5 and SbbHLH6 did not demonstrate interactions with other SbbHLH proteins, suggesting that these two genes may be involved in distinct metabolic pathways.

Gene expression patterns can offer valuable insights into gene function. The bHLH gene family displays considerable variation in abundance among different organisms and tissues, contributing to diverse physiological roles [36]. In our research, the SbbHLH genes displayed distinct expression patterns. Some SbbHLHs showed peak transcript levels at specific stages of reproductive development, suggesting that they may regulate particular developmental processes by influencing various target genes. Our findings revealed that the expression levels of *SbbHLH3*, *SbbHLH8*, and *SbbHLH9* were dramatically upregulated (over 20-fold in qPCR results) in the primordia or fruiting bodies, and thus we speculated that these three genes participate in development processes of *S. baumii* (Figure 6). Additionally, numerous studies have indicated that bHLH genes are crucial in responding to abiotic stress [21,38,39,40]. The increased expression of *SbbHLHs* under heat stress suggests their extensive involvement in the heat stress response. Among the identified 12 *SbbHLHs*, *SbbHLH3* and *SbbHLH9* exhibited enhanced expression during the reproductive stages and under various abiotic stress conditions, highlighting their multifunctional roles in *S. baumii* (Figure 7). Notably, *SbbHLH3* exhibited particularly high expression levels across different treatments. Consequently, heterologous expression in yeast was utilized in subsequent experiments to explore the preliminary functions of *SbbHLH3*. The findings demonstrated that the overexpression of *SbbHLH3* significantly improved the tolerance of yeast cells to various stresses (Figure 8, Figure 9 and Figure 10). This suggests that *SbbHLH3* may play a positive role in enhancing the resilience of *S. baumii* to abiotic stresses.

This study has some limitations, such as the inability to confirm protein–protein interactions in vivo. In addition, the specific mechanism by which the *SbbHLH3* gene contributes to the resistance of *S. baumii* was not fully investigated, even though the *bHLH* gene family was identified. Future research should aim to explore the resistance-related genes in *S. baumii*, and analyze the relationship between its medicinal properties and resistance. Furthermore, gaining insights into the molecular characteristics of *S. baumii* will enhance its economic potential.

## 5. Conclusions

In summary, a thorough analysis identified 12 *SbbHLH* genes in the *S. baumii* genome. These genes underwent various evaluations, including physicochemical properties, phylogenetic relationships, conserved motifs, gene structure, cis-regulatory element, protein–protein interactions, and expression patterns. Notably, the expression analysis and heterologous expression assays in yeast indicated that *SbbHLH3* may be involved in development and abiotic stress responses in *S. baumii*. This study provides detailed insights into the functional mechanisms and expression profiles of *SbbHLHs* in *S. baumii*, laying the theoretical groundwork for future research on the molecular mechanisms of *SbbHLHs*.

## Figures and Tables

**Table 1:** The 12 bHLH transcription factors identified in *S. baumii*.

No.	Gene	Gene ID	CDS Length (bp)	Protein Size (aa)	Molecular Weight (kDa)	pI	Predicted Localization
1	*SbbHLH1*	*A7U60_g1820*	912	303	32.59	6.48	nuclear
2	*SbbHLH2*	*A7U60_g1898*	1881	626	67.93	9.55	nuclear
3	*SbbHLH3*	*A7U60_g2241*	2589	862	89.35	6.38	nuclear
4	*SbbHLH4*	*A7U60_g2862*	1140	379	41.10	5.97	nuclear
5	*SbbHLH5*	*A7U60_g3672*	1716	571	61.27	8.03	extracellular
6	*SbbHLH6*	*A7U60_g4654*	735	244	26.26	6.44	nuclear
7	*SbbHLH7*	*A7U60_g6711*	1374	457	48.07	6.08	nuclear
8	*SbbHLH8*	*A7U60_g7205*	3765	1254	137.86	6.39	mitochondrial
9	*SbbHLH9*	*A7U60_g7247*	915	304	33.74	7.91	nuclear
10	*SbbHLH10*	*A7U60_g7248*	1050	349	38.75	6.23	nuclear
11	*SbbHLH11*	*A7U60_g8019*	3498	1165	125.74	5.50	nuclear
12	*SbbHLH12*	*A7U60_g8101*	2796	931	96.97	6.17	nuclear

## Data Availability

The original contributions presented in this study are included in the article/Supplementary Materials. Further inquiries can be directed to the corresponding author.

## Supplementary Materials

The following supporting information can be downloaded at: https://www.mdpi.com/article/10.3390/genes16020184/s1, Figure S1: The three-dimensional (3D) architecture of SbbHLH proteins; Figure S2: PCR gel electrophoresis identification results for the recombinant yeast INVSC1 (pYES2-Sb-bHLH3) strains. Table S1: Primers used for this study; Table S2: List of the 12 *SbbHLH* genes identified in this study; Table S3: Analysis and distribution of conserved motifs in *S. baumii* bHLH proteins; Table S4: List of the bHLH genes of six macrofungus species identified in this study; Table S5: Analysis and distribution of conserved motifs in bHLH proteins of seven macrofungus species.

## References

[1] Zou L., Sun T.T., Li D.L., Tan Y., Zhang G.Q., Wang F., Zhang J. (2016). De novo transcriptome analysis of *Inonotus baumii* by RNA-seq. J. Biosci. Bioeng..

[2] Wang S., Liu Z., Wang X., Liu R., Zou L. (2022). Mushrooms Do Produce Flavonoids: Metabolite Profiling and Transcriptome Analysis of Flavonoid Synthesis in the Medicinal Mushroom *Sanghuangporus baumii*. J. Fungi.

[3] Qu Y.H., Zhang P.P., Cui J., Ni X.Z., Song K., Shi D.F. (2024). Extraction Optimization, Structure Analysis and Antioxidant Activity of Polysaccharide from *Sanghuangporus Baumii*. Curr. Anal. Chem..

[4] Chien L.H., Deng J.S., Jiang W.P., Chen C.C., Chou Y.N., Lin J.G., Huang G.J. (2022). Study on the potential of *Sanghuangporus sanghuang* and its components as COVID-19 spike protein receptor binding domain inhibitors. Biomed. Pharmacother..

[5] Liu M.M., Zeng P., Li X.T., Shi L.G. (2016). Antitumor and immunomodulation activities of polysaccharide from *Phellinus Baumii*. Int. J. Biol. Macromol..

[6] Wang H., Ma J.X., Zhou M., Si J., Cui B.K. (2022). Current advances and potential trends of the polysaccharides derived from medicinal mushrooms sanghuang. Front. Microbiol..

[7] Liu Z., Tong X., Liu R., Zou L. (2022). Metabolome and Transcriptome Profiling Reveal That Four Terpenoid Hormones Dominate the Growth and Development of *Sanghuangporus baumii*. J. Fungi.

[8] Yang Q.Q., Feng K., Xu Z.S., Duan A.Q., Liu J.X., Xiong A.S. (2019). Genome-wide identification of bZIP transcription factors and their responses to abiotic stress in celery. Biotechnol. Biotechnol. Equip..

[9] Sun X., Wang Y., Sui N. (2018). Transcriptional regulation of bHLH during plant response to stress. Biochem. Biophys. Res. Commun..

[10] Yan T., Shu X., Ning C., Li Y., Wang Z., Wang T., Zhuang W. (2024). Functions and Regulatory Mechanisms of bHLH Transcription Factors during the Responses to Biotic and Abiotic Stresses in Woody Plants. Plants.

[11] Schwechheimer C., Zourelidou M., Bevan M.W. (1998). Plant transcription factor studies. Annu. Rev. Plant Physiol. Plant Mol. Biol..

[12] Ledent V., Vervoort M. (2001). The basic helix-loop-helix protein family: Comparative genomics and phylogenetic analysis. Genome Res..

[13] Ma P.C., Rould M.A., Weintraub H., Pabo C.O. (1994). Crystal structure of MyoD bHLH domain-DNA complex: Perspectives on DNA recognition and implications for transcriptional activation. Cell.

[14] Shimizu T., Toumoto A., Ihara K., Shimizu M., Kyogoku Y., Ogawa N., Oshima Y., Hakoshima T. (1997). Crystal structure of PHO4 bHLH domain-DNA complex: Flanking base recognition. EMBO J..

[15] Ludwig S.R., Habera L.F., Dellaporta S.L., Wessler S.R. (1989). Lc, a member of the maize R gene family responsible for tissue-specific anthocyanin production, encodes a protein similar to transcriptional activators and contains the myc-homology region. Proc. Natl. Acad. Sci. USA.

[16] Carretero-Paulet L., Galstyan A., Roig-Villanova I., Martínez-García J.F., Bilbao-Castro J.R., Robertson D.L. (2010). Genome-wide classification and evolutionary analysis of the bHLH family of transcription factors in Arabidopsis, poplar, rice, moss, and algae. Plant Physiol..

[17] Fan Y., Yang H., Lai D.L., He A.L., Xue G.X., Feng L., Chen L., Cheng X.B., Ruan J.J., Yan J. (2021). Genome-wide identification and expression analysis of the bHLH transcription factor family and its response to abiotic stress in sorghum [*Sorghum bicolor* (L.) Moench]. BMC Genom..

[18] Dong J., Wu Y.W., Dong Y., Pu R., Li X.J., Lyu Y.M., Bai T., Zhang J.L. (2024). Genome-Wide Identification of the bHLH Gene Family in *Rhododendron delavayi* and Its Expression Analysis in Different Floral Tissues. Genes.

[19] Yang M., Zhou C., Yang H., Kuang R., Huang B., Wei Y. (2020). Genome-wide analysis of basic helix-loop-helix transcription factors in papaya (*Carica papaya* L.). PeerJ.

[20] Qiao X., Li M., Li L., Yin H., Wu J.Y., Zhang S.L. (2015). Genome-wide identification and comparative analysis of the heat shock transcription factor family in Chinese white pear (*Pyrus bretschneideri*) and five other Rosaceae species. BMC Plant Biol..

[21] Chen Y.Y., Li M.Y., Wu X.J., Huang Y., Ma J., Xiong A.S. (2015). Genome-wide analysis of basic helix-loop-helix family transcription factors and their role in responses to abiotic stress in carrot. Mol. Breed..

[22] Zhou X., Liao Y., Kim S.U., Chen Z., Nie G., Cheng S. (2020). Genomewide identification and characterization of bHLH family genes from *Ginkgo biloba*. Sci. Rep..

[23] Wang L.N., Gao W., Wu X.L., Zhao M.R., Qu J.B., Huang C.Y., Zhang J.X. (2018). Genome-Wide Characterization and Expression Analyses of *Pleurotus ostreatus* MYB Transcription Factors during Developmental Stages and under Heat Stress Based on *de novo* Sequenced Genome. Int. J. Mol. Sci..

[24] Ohm R.A., de Jong J.F., de Bekker C., Wösten H.A., Lugones L.G. (2011). Transcription factor genes of *Schizophyllum commune* involved in regulation of mushroom formation. Mol. Microbiol..

[25] Potter S.C., Luciani A., Eddy S.R., Park Y., Lopez R., Finn R.D. (2018). HMMER web server: 2018 update. Nucleic Acids Res..

[26] Letunic I., Bork P. (2018). 20 years of the SMART protein domain annotation resource. Nucleic Acids Res..

[27] Hu B., Jin J.P., Guo A.Y., Zhang H., Luo J.C., Gao G. (2014). GSDS 2.0: An upgraded gene feature visualization server. Bioinformatics.

[28] Kumar S., Stecher G., Li M., Knyaz C., Tamura K. (2018). MEGA X: Molecular evolutionary genetics analysis across computing platforms. Mol. Biol. Evol..

[29] Chen C., Chen H., Zhang Y., Thomas H.R., Frank M.H., He Y., Xia R. (2020). TBtools: An integrative toolkit developed for interactive analyses of big biological data. Mol. Plant.

[30] Bailey T.L., Boden M., Buske F.A., Frith M., Grant C.E., Clementi L., Ren J., Li W.W., Noble W.S. (2009). MEME SUITE: Tools for motif discovery and searching. Nucleic Acids Res..

[31] Lescot M., Déhais P., Thijs G., Marchal K., Moreau Y., Van de Peer Y., Rouzé P., Rombauts S. (2002). PlantCARE, a database of plant cis-acting regulatory elements and a portal to tools for in silico analysis of promoter sequences. Nucleic Acids Res..

[32] Szklarczyk D., Gable A.L., Lyon D., Junge A., Wyder S., Huerta-Cepas J., Simonovic M., Doncheva N.T., Morris J.H., Bork P. (2019). STRING v11: Protein-protein association networks with increased coverage, supporting functional discovery in genome-wide experimental datasets. Nucleic Acids Res..

[33] Liu Z., Liu R., Tong X., Zou L. (2022). New Insights into Methyl Jasmonate Regulation of Triterpenoid Biosynthesis in Medicinal Fungal Species *Sanghuangporus baumii* (Pilát) L.W. Zhou & Y.C. Dai. J. Fungi.

[34] Livak K.J., Schmittgen T.D. (2001). Analysis of relative gene expression data using real-time quantitative PCR and the 2^−∆∆CT^ method. Methods.

[35] Ji X.L., Yang F., Zhou X.M., Jia W.Q., Zhu X.P., Mu J.Y., Wang Y.L., Zhang Y., Mi Z.R., Zhang S.L. (2025). Genome-wide identification of the bHLH gene family and the mechanism regulation of anthocyanin biosynthesis by ChEGL1 in *Cerasus humilis*. Int. J. Biol. Macromol..

[36] Zhang T.T., Lv W., Zhang H.S., Ma L., Li P.H., Ge L., Li G. (2018). Genome-wide analysis of the basic HelixLoop-Helix (bHLH) transcription factor family in maize. BMC Plant Biol..

[37] Li Y.Y., Sui X.Y., Yang J.S., Xiang X.H., Li Z.Q., Wang Y.Y. (2020). A novel bHLH transcription factor, NtbHLH1, modulates iron homeostasis in tobacco (*Nicotiana tabacum* L.). Biochem. Biophys. Res. Commun..

[38] Lu X.W., Zhang H., Hu J.L., Nie G., Khan I., Feng G.Y., Zhang X.Q., Wang X.S., Huang L.K. (2022). Genome-wide identification and characterization of bHLH family genes from orchardgrass and the functional characterization of *DgbHLH46* and *DgbHLH128* in drought and salt tolerance. Funct. Integr. Genom..

[39] Qian Y., Zhang T., Yu Y., Gou L., Yang J., Xu J., Pi E. (2021). Regulatory Mechanisms of bHLH Transcription Factors in Plant Adaptive Responses to Various Abiotic Stresses. Front. Plant Sci..

[40] Li T., Shi Y., Zhu B., Zhang T., Feng Z., Wang X., Li X., You C. (2022). Genome-Wide Identification of Apple Atypical bHLH Subfamily PRE Members and Functional Characterization of MdPRE4.3 in Response to Abiotic Stress. Front. Genet..

